# Different body parts’ fat mass and corrected QT interval on the electrocardiogram: The Fasa PERSIAN Cohort Study

**DOI:** 10.1186/s12872-021-02095-2

**Published:** 2021-06-05

**Authors:** Mohammad Hosein Yazdanpanah, Ehsan Bahramali, Mohammad Mehdi Naghizadeh, Mojtaba Farjam, Maryam Mobasheri, Shiva Dadvand

**Affiliations:** 1 Student Research Committee, Fasa University of Medical Sciences, Fasa, Iran; 2 Noncommunicable Diseases Research Center, Fasa University of Medical Sciences, Fasa, Iran; 3 Non-communicable Diseases Research Center, Fars Heart Foundation, Kowsar Hospital, Shiraz, Iran

**Keywords:** Fat mass, Fat mass to fat-free mass (FM/FFM) ratio, QT interval, QTc prolongation, Cardio-metabolic, Obesity, Electrocardiogram, Iran, Load-capacity model

## Abstract

**Background:**

Previous studies suggested that obesity and fat mass are associated with QT interval prolongation, but the role of different body parts' fat mass is unclear. The associations between total and regional fat mass (FM) and corrected QT interval (QTc) were investigated for the first time in this study.

**Methods:**

In this sub-analysis of Fasa PERSIAN cohort Study data, 3217 subjects aged 35–70 entered our study. Body fat mass was assessed by bioelectrical impedance analysis and QTc interval calculated by the QT interval measured by Cardiax^®^ software from ECGs and Bazett’s formula. Uni- and multi-variable linear and logistic regression was performed in IBM SPSS Statistics v23.

**Results:**

In males, the fat mass to fat-free mass (FM/FFM) ratio in the trunk, arms, total body, and legs were significantly higher in the prolonged QTc group (QTc > 450 ms). Trunk (B = 0.148), total (B = 0.137), arms (B = 0.124), legs (B = 0.107) fat mass index (FMI) showed significant positive relationship with continuous QTc (P-value < 0.001). Also, just the fat-free mass index of legs had significant positive associations with QTc interval (P-value < 0.05). Surprisingly, in females, the mean of FM/FFM ratio in trunk and legs in the normal QTc group had higher values than the prolonged QTc group (QTc > 470 ms). Also, none of the body composition variables had a significant correlation with continuous QTc.

**Conclusion:**

Our study suggested that FMI ratios in the trunk, total body, arms, and legs were positively associated with QTc interval in males, respectively, from a higher to a lower beta-coefficient. Such associations were not seen in females. Our study implies that body fat mass may be an independent risk factor for higher QTc interval and, consequently, more cardiovascular events that should be investigated.

**Supplementary Information:**

The online version contains supplementary material available at 10.1186/s12872-021-02095-2.

## Introduction

Total depolarization and repolarization times of cardiomyocytes are reflected in the QT interval in the surface electrocardiogram (ECG). Thus, any alteration in the QT interval can indicate abnormalities in the two phases of the cardiomyocyte electrical cycle. Any QT prolongation is primarily secondary to a prolonged repolarization period, and it may be congenital or acquired. Some of the known and suggested risk factors of prolonged QT are age [1], female gender [2], smoking [3], hyperlipidemia [4] and a history of prior cardiovascular diseases [5], metabolic syndrome [6], and comorbidities like diabetes, renal failure, thyroid disturbances [7], and liver failure [8]. Electrolytes like potassium [9] and calcium [7] have immediate and long-term effects on the QT interval; however, it is more of a concern in acute settings. Also, there is a long list of drugs that affect QT, some of which can produce arrhythmia [10].

One of the most prevalent cardiovascular risk factors which can affect QT interval, directly and indirectly, is obesity. Increasing body mass index (BMI) can alter the heart rate and, thus, the corrected QT [2, 3]. Obesity is also associated with other comorbidities and cardiovascular diseases (CVD) risk factors, including diabetes, hypertension, and hyperlipidemia. Obesity can lead to increased morbidity and mortality due to CVD as important NCDs among the Iranian population [11–13]. A previous study in an Iranian population showed that central adiposity is a significant determinant in CVD risk [14]. Also, in the Framingham study, obesity was one of the significant causes of mortality due to heart disease [15].

Compelling evidence indicates that the QT interval is longer in overweight or obese individuals than in the average population [16]. Interestingly, after weight loss, either through diet and exercise or through surgery, the QT interval shortens [16, 17]. Making it more quantitative, Carella et al. showed a 5-ms increase in QT interval by every 50% increase in fat mass percentage measured by hydrodensitometry. The health effect of fat mass is not about the fat volume itself but metabolic, neuroendocrine, and inflammatory changes linked to fat mass and its regional depots. Some of the suggested fat mass effect mechanisms include impaired fasting glucose [18], hyperinsulinemia and insulin resistance [19], HR-variability, and SNS activity [20].

Longer QT intervals have historically been believed to be a risk factor for developing arrhythmia in normal and diseased hearts [21]. The QT interval has served as a prognostic factor in specific subgroups of patients, including those with ischemic heart disease or heart failure. Other measures related to it, like QT dispersion, have received much attention as predictors of sudden death in these patient populations [22]. The heterogeneity of cardiomyocyte electrical activity, reflected in surface ECG as QT interval, explains why QT interval can discriminate an arrhythmia-prone heart and consequently serves as a predictive tool [23].

Determining body composition and distribution of fat in body parts has been made possible using various devices that introduce small electrical currents through different routes in the corpus and measures the electrical impedance accordingly. Body composition as a reliable way for distinguishing the fat disturbances in various body parts was used in our study to seek which body part's fat mass is associated with corrected QT (QTc) interval in an Iranian population.

## Methods

### Study population

In this cross-sectional study, we used collected data of Fasa Cohort Study, a branch of Prospective Epidemiological Research Studies in IrAN (PERSIAN) [24]. This study is a prospective population-based cohort to evaluate non-communicable diseases (NCDs) in participants aged 35–70 with a 15-year follow-up. Before registration in Fasa Cohort Study, informed consent is obtained from each participant. All subjects with available data of both body composition and electrocardiograms (n = 3311) entered our study. Nighty-four subjects (2.83%) were excluded due to lack of data of ECG and Body composition, non-sinus rhythm, wide QRS complex (QRS duration > 120 ms), which can falsely increase QT interval. Eventually, 3217 subjects (1415 male, 1802 female) remained in the study. Demographic data, including age, sex was recorded in a questionnaire. Also, the current status of smoking has been questioned: "Do you smoke cigarettes? Yes or no”. NCDs' history, such as diabetes and hypertension, and coronary heart disease (CHD), have been questioned and recorded. Diabetes was determined from self-reported history, use of oral hypoglycemics, or insulins. Coronary heart disease was determined by the self-reported history of coronary artery bypass graft surgery, coronary angioplasty, myocardial infarction, angina, or ECG evidence of myocardial infarction by identifying major Q-wave. Medication taken within two weeks was asked to be brought for registration at interview time for maximum precision. CredibleMeds, as an American organization, provides lists of drugs associated with QTc-prolongation, which is continuously updated by their review team based on new information. We used a list of including drugs with a known risk for TdP and medications with a possible risk for TdP, which counts 187 cardiac and non-cardiac drugs in total [25].

### Measurements

For serum total and high-density lipoprotein cholesterol (TC and HDL) assessment, venous blood samples were taken in a 10-14 h fasting condition. The sampling was performed by trained personnel and transported to the laboratory in standard condition. TC and HDL measures were assayed using the Mindray BS380 autoanalyzer (Mindray Medical International, Shenzhen, China) for the biochemical tests. A stadiometer measured height with an accuracy of 0.1 cm, and a digital scale measured weight with an accuracy of 0.1 kg. Body mass index (BMI) was calculated using a value divided by the square of height (kg/m^2^). A nurse was in charge of taking resting blood pressure. Systolic and diastolic blood consists of 2 measurements in the seating position in 15 min and is reported by an average pressure in mmHg.

### Body composition

Bioelectric impedance analysis (BIA) was used for measuring body composition using the Tanita BC-418 MA Segmental Body Composition Analyzer (Tanita, Japan). This device is a single-frequency BIA device with eight polar electrodes and a single-point load cell weighing system in the scale platform that can provide separate body mass readings for various body segments, including the right arm, left arm, trunk, right leg, and left leg. The impedance across the subject's tissues is determined with receiver electrodes after a predefined signal is passed through injector electrodes. All measurements are performed at 50 kHz with a steady current of 0.8 mA sine wave. FM percent is calculated using an algorithm that takes into account impedance, age, and height. Body composition assessment was reported by arm fat mass (AFM), arms fat-free mass (AFFM), legs fat mass (LFM), legs fat-free mass (LFFM), trunk fat mass (TFM), truncal fat-free mass (TFFM), total fat mass (FM) and total fat-free mass (FFM). Fat mass percentages in total and regional depots were also calculated. The fat(-free) mass index (FMI) was calculated using fat(-free) mass (total or regional depots) in Kg divided by the square of height in m (kg/m2). Also, the fat mass to fat-free mass ratio (FM/FFM) has been calculated for total and different body regions. These ratios are representing the load-capacity model, following Siervo et al. [26]. FM has a negative influence on physiological function, while FFM has a beneficial effect in this model.

### Electrocardiogram

Each participant had 12-lead electrocardiograms (ECG) with a 2000 Hz sampling rate and 0.04 µV/bit (24-bit resolution). ECGs were recorded by trained personnel using a computer-based device (Cardiax^®^) [27], and the recording was performed on shaved precordium in the supine and postprandial state for the best possible results. Heart rates and QT intervals (Lead II) of all ECGs were reported automatically by Cardiax software (version 3.50.2, International Medical Equipment Developing Co. Ltd., Budapest, Hungary) and exported to central data software. Also, QTc was calculated by Bazett’s formula (QTc = QT/(RR)^1/2^) [28]; Bazett's formula was used due to widespread use in clinical situations and having the standardized clinical cut-offs. Based on clinical standards, QTc < 450 ms for males and QTc < 470 ms for females was considered Normal QTc, higher QTc than these two cut-points considered as prolonged QTc in both genders [29, 30].

### Statistical analyses

All variables were reported as Mean ± standard deviation (SD), and number (Percentage %). There was not any missing data among the raw data. For comparison between the two groups, we used an independent-samples t-test. To determine the correlation between body composition and continuous QTc, linear regression was used, also with dichotomous QTc (Normal and Prolonged), logistic regression was used. The regression standardized beta coefficient (B) with its P-value in the first analysis and the OR and 95% Confidence intervals (95%CI) with its p-value in the last analysis were reported. About regression analysis were adjusted by conventional CVD risk factors, including age (years), smoking status (yes = 1), total cholesterol (mg/dL), high-density lipoprotein (mg/dL), and blood pressure (mmHg), history of CHD (yes = 1), diabetes (yes = 1), and selected medication as drugs known for prolonging QT interval (consume = 1). More details of drugs were mentioned above25. Also, 5th, 15th, 25th, 50th, 75th, 85th, and 95th percentiles for FM/FFM ratio have been calculated in both genders. Significant P-value was considered at the P-value level < 0.05, and all analysis was performed using IBM SPSS Statistics, version 23 (IBM Corp., Armonk, N.Y., USA). Also, for graphs, we used Prism version 8.00 (GraphPad Software, La Jolla California, USA).

## Results

In a total of 3217 participants, the mean age was 47.39 ± 9.23 years in men and 48.12 ± 9.55 years in women. The mean BMI was 23.96 ± 4.50 kg/m^2^ in men and 27.03 ± 4.97 kg/m^2^ in women. There were 794(56.1%) male subjects, and 68(3.8%) of the females reported active smoking. In males, 104(7.3%) and 302(16.8%) reported diabetes in females. The mean of QT interval before and after correction respectively in men was 403.33 ± 37.72 ms and 418.00 ± 32.85 ms, and in women was 396.61 ± 42.38 ms and 438.65 ± 39.77 ms. The difference between males and females in QT and corrected QT intervals was statistically significant (P-value < 0.001). Other variables mean and SD, number (%) according to gender reported in Table 1.

**Table 1:** Baseline characteristic of subjects according to gender

Variables	Male (n = 1415)	Female (n = 1802)	P-value
Age (years)	47.39 ± 9.23	48.12 ± 9.55	**0.030**^**a**^
BMI (kg/m^2^)	24.19 ± 4.41	26.86 ± 4.81	**< 0.001**^**a**^
SBP (mmHg)	108.31 ± 16.99	109.35 ± 18.56	0.097^a^
DBP (mmHg)	72.43 ± 11.85	72.46 ± 11.94	0.933^a^
TC (mg/dL)	178.13 ± 38.64	189.01 ± 39.25	**< 0.001**^**a**^
HDL (mg/dL)	43.49 ± 9.16	49.62 ± 10.31	**< 0.001**^**a**^
Current smoker	794 (56.1)	68 (3.8)	**< 0.001**^**b**^
Coronary heart disease	80 (5.7)	206 (11.4)	**< 0.001**^**b**^
Diabetes mellitus	104 (7.3)	302 (16.8)	**< 0.001**^**b**^
Hypertension	141 (10.0)	497 (27.6)	**< 0.001**^**b**^
Selected medications	965 (68.2)	1195 (66.3)	0.259^b^
Heart rate (bpm)	65.61 ± 10.79	74.51 ± 11.76	**< 0.001**^**a**^
QRS duration (ms)	105.31 ± 9.23	90.87 ± 6.35	**< 0.001**^**a**^
QT interval (ms)	403.33 ± 37.72	396.61 ± 42.38	**< 0.001**^**a**^
Bazett's QTc interval (ms)	418.53 ± 32.85	438.65 ± 39.77	**< 0.001**^**a**^
Fridericia's QTc interval (ms)	413.12 ± 30.93	423.92 ± 37.95	**< 0.001**^**a**^

Data presented as mean ± standard deviation, number (frequency)

BMI = body mass index, SBP = systolic blood pressure, DBP = Diastolic blood pressure, TC = Total cholesterol, HDL = High density lipoprotein, QTc = corrected QT interval

P-value reported as the result of ^a^independent-samples t-test and ^b^Chi-square. Statistically significant P-values are bolded (P-value < 0.05)

Table 2 shows the means of total and regional body composition data and BMI in total, normal QTc, and prolonged QTc interval groups according to gender. Total FMI was 4.97 ± 2.55 kg/m^2^ and 9.56 ± 3.45 kg/m^2^ in the total male and female population, respectively. Total FMI and truncal FMI were higher in males with prolonged QTc and showed a significant difference between normal and prolonged QT groups (P-value < 0.05), while it was higher in females with normal QTc, it did not show any significant difference between groups. Both fat percentage and FM/FFM ratio in total, arms, and trunk showed a significant difference between QT interval groups in males. Also, in females, truncal fat percentage and FM/FFM ratio showed a significant difference between QT interval groups, but their mean was higher in the normal QT group than in a prolonged QT group(P-value < 0.05).

**Table 2:** The mean and standard deviation of total and regional body composition data and BMI in total, normal QTc and prolonged QTc interval groups according to gender

	Male				Female			
	Total (n = 1415)	QTc ≤ 450 ms (n = 1281)	QTc > 450 ms (n = 134)	P-value	Total (n = 1802)	QTc ≤ 470 ms (n = 1621)	QTc > 470 ms (n = 181)	P-value
*Total*								
Fat mass (%)	19.50 ± 7.10	19.35 ± 7.04	20.92 ± 7.50	**0.015**	34.11 ± 6.99	34.23 ± 6.89	33.07 ± 7.72	**0.034**
FMI (kg/m^2^)	4.97 ± 2.56	4.93 ± 2.53	5.43 ± 2.73	**0.028**	9.57 ± 3.46	9.61 ± 3.44	9.22 ± 3.59	0.153
FFMI (kg/m^2^)	19.17 ± 2.26	19.18 ± 2.25	19.15 ± 2.30	0.893	17.61 ± 1.80	17.61 ± 1.79	17.63 ± 1.82	0.883
FM:FFM ratio	0.25 ± 0.11	0.25 ± 0.11	0.28 ± 0.12	**0.009**	0.53 ± 0.16	0.54 ± 0.16	0.51 ± 0.17	0.056
*Arms*								
Fat mass (%)	35.63 ± 11.43	35.36 ± 11.33	38.25 ± 12.10	**0.005**	70.75 ± 18.18	71.00 ± 17.99	68.47 ± 19.66	0.075
FMI (kg/m^2^)	0.50 ± 0.27	0.50 ± 0.27	0.54 ± 0.28	0.056	1.07 ± 0.52	1.07 ± 0.52	1.03 ± 0.53	0.279
FFMI (kg/m^2^)	2.15 ± 0.35	2.15 ± 0.35	2.13 ± 0.36	0.490	1.78 ± 0.23	1.78 ± 0.23	1.78 ± 0.23	0.910
FM:FFM ratio	0.22 ± 0.09	0.22 ± 0.09	0.24 ± 0.10	**0.005**	0.58 ± 0.22	0.58 ± 0.22	0.55 ± 0.23	0.121
*Legs*								
Fat mass (%)	33.58 ± 11.50	33.42 ± 11.43	35.12 ± 12.08	0.102	80.65 ± 10.16	80.76 ± 10.08	79.63 ± 10.82	0.155
FMI (kg/m^2^)	1.40 ± 0.70	1.39 ± 0.70	1.49 ± 0.78	0.117	4.06 ± 1.19	4.07 ± 1.19	3.98 ± 1.22	0.340
FFMI (kg/m^2^)	6.53 ± 0.86	6.52 ± 0.86	6.56 ± 0.91	0.679	5.82 ± 0.67	5.82 ± 0.67	5.81 ± 0.69	0.745
FM:FFM ratio	0.21 ± 0.09	0.21 ± 0.09	0.22 ± 0.09	0.092	0.69 ± 0.14	0.69 ± 0.14	0.67 ± 0.15	0.183
*Trunk*								
Fat mass (%)	21.46 ± 8.33	21.27 ± 8.26	23.29 ± 8.75	**0.007**	29.59 ± 8.34	29.75 ± 8.24	28.20 ± 9.10	**0.018**
FMI (kg/m^2^)	3.09 ± 1.62	3.05 ± 1.60	3.41 ± 1.70	**0.014**	4.45 ± 1.82	4.47 ± 1.81	4.22 ± 1.90	0.076
FFMI (kg/m^2^)	10.49 ± 1.12	10.50 ± 1.12	10.46 ± 1.12	0.702	10.02 ± 0.96	10.02 ± 0.97	10.05 ± 0.95	0.659
FM:FFM ratio	0.29 ± 0.14	0.28 ± 0.13	0.32 ± 0.14	**0.004**	0.44 ± 0.17	0.44 ± 0.17	0.41 ± 0.17	**0.029**
BMI (kg/m^2^)	24.19 ± 4.41	23.91 ± 4.47	24.47 ± 4.80	0.170	26.86 ± 4.81	27.07 ± 4.96	26.6 ± 5.12	0.329

QTc = corrected QT interval by Bazett formula. P-value reported as the result of independent-samples t-test between normal and prolonged QTc interval groups. Statistically significant P-values are bolded (P-value< 0.05)

In males, AFM, TFM, and Total FM were significantly higher in the prolonged QTc group compared to Normal QTc (P-value < 0.05); although LFM was higher in the prolonged QTc group, it was not significant (P-value = 0.105). In females, AFM, LFM, TFM, and Total FM were more elevated in the normal QTc group, although they were in contrast to male results as they were not statistically significant (P-value > 0.05). Also, none of the FFMs showed a considerable difference between QTc groups in both genders.

BMI did not show any significant difference between normal QTc and prolonged QTc interval groups in both genders (P-value > 0.05). BMI was higher in the male prolonged QTc group than the male normal QTc group, but it was not significant (P-value = 0.170). In females, higher BMI was observed in the normal QTc group, but it had not shown a significant difference between the QTc group (P-value = 0.329). Also, the mean heart rate was 64.77 ± 10.09 bpm, 74.18 ± 11.68 bpm in normal and 73.69 ± 13.61 bpm, 77.48 ± 12.09 bpm in prolonged QTc group respectively in male and female, which was a significant difference (P-value < 0.001).

In male, Arms (B = 0.158), Legs (B = 0.104), Trunk (B = 0.152) and total (B = 0.142) FM percentage showed significant positive relationship with continuous QTc (P-value < 0.001). Also, FMI and FM/FFM ratio in total and all regions had significant positive associations with continuous QTc (P-value < 0.001). The highest beta-coefficient of FMI was related to trunk (B = 0.148), followed by total (B = 0.137). All of these relationships remained significant after multi-variable adjust in linear regression (P-value < 0.01). In logistic regression, after multivariable-adjusting, FM/FFM ratios in total, trunk, and arms showed significant associations with QTc > 450 ms with ORs 6.66, 5.58, and 11.31, respectively (P-value < 0.05). In fat percentages, just arms and trunk showed significant associations (P-value < 0.05). All B coefficients, ORs, and their P-values are reported as the results of both unadjusted and multivariable-adjusted regression in Table 3 for males and Additional File 1: Table A1 for females.

**Table 3:** The association between total and regional body composition data and QTc interval in male

	Unadjusted				Multi-variable adjusted			
	QTc (continuous)		QTc > 450 ms		QTc (continuous)		QTc > 450 ms	
	Beta	P-value	OR (95%CI)	P-value	Beta	P-value	OR (95%CI)	P-value
*Total*								
Fat mass (%)	0.142	**< 0.001**	1.03 (1.01–1.06)	**0.015**	0.125	**< 0.001**	1.03 (1.00–1.06)	0.057
FMI (kg/m^2^)	0.137	**< 0.001**	1.08 (1.01–1.15)	**0.029**	0.122	**< 0.001**	1.07 (0.99–1.15)	0.078
FFMI (kg/m^2^)	0.038	0.154	1.00 (0.92–1.08)	0.893	0.029	0.331	0.99 (0.91–1.09)	0.885
FM:FFM ratio	0.149	**< 0.001**	8.32 (1.70–40.73)	**0.009**	0.131	**< 0.001**	6.66 (1.11–40.13)	**0.038**
*Arms*								
Fat mass (%)	0.158	**< 0.001**	1.02 (1.01–1.04)	**0.005**	0.145	**< 0.001**	1.02 (1.00–1.04)	**0.018**
FMI (kg/m^2^)	0.124	**< 0.001**	1.74 (0.98–3.09)	0.060	0.113	**< 0.001**	1.73 (0.92–3.27)	0.090
FFMI (kg/m^2^)	0.008	0.773	0.84 (0.50–1.39)	0.490	0.007	0.813	0.88 (0.49–1.57)	0.659
FM:FFM ratio	0.157	**< 0.001**	12.61 (2.09–76.09)	**0.006**	0.142	**< 0.001**	11.31 (1.51–84.94)	**0.018**
*Legs*								
Fat mass (%)	0.104	**< 0.001**	1.01 (1.00–1.03)	0.102	0.076	**0.0101**	1.01 (0.99–1.03)	0.271
FMI (kg/m^2^)	0.107	**< 0.001**	1.21 (0.95–1.54)	0.117	0.090	**0.002**	1.19 (0.91–1.56)	0.202
FFMI (kg/m^2^)	0.064	**0.015**	1.04 (0.85–1.28)	0.679	0.081	**0.007**	1.09 (0.87–1.38)	0.443
FM:FFM ratio	0.106	**< 0.001**	5.55 (0.76–40.64)	0.092	0.079	**0.008**	3.87 (0.40–37.84)	0.245
*Trunk*								
Fat mass (%)	0.152	**< 0.001**	1.03 (1.01–1.05)	**0.008**	0.137	**< 0.001**	1.03 (1.00–1.05)	**0.032**
FMI (kg/m^2^)	0.148	**< 0.001**	1.14 (1.03–1.27)	**0.014**	0.132	**< 0.001**	1.12 (1.00–1.27)	0.053
FFMI (kg/m^2^)	0.025	0.346	0.97 (0.83–1.14)	0.702	− 0.004	0.900	0.94 (0.78–1.12)	0.457
FM:FFM ratio	0.160	**< 0.001**	6.76 (1.84–24.79)	**0.004**	0.145	**< 0.001**	5.58 (1.31–23.72)	**0.020**

B = beta coefficient, QTc = Corrected QT interval by Bazett's formula, OR = Odds ratio, CI = Confidence interval. Statistically significant P-values are bolded

**Fig. 1:**
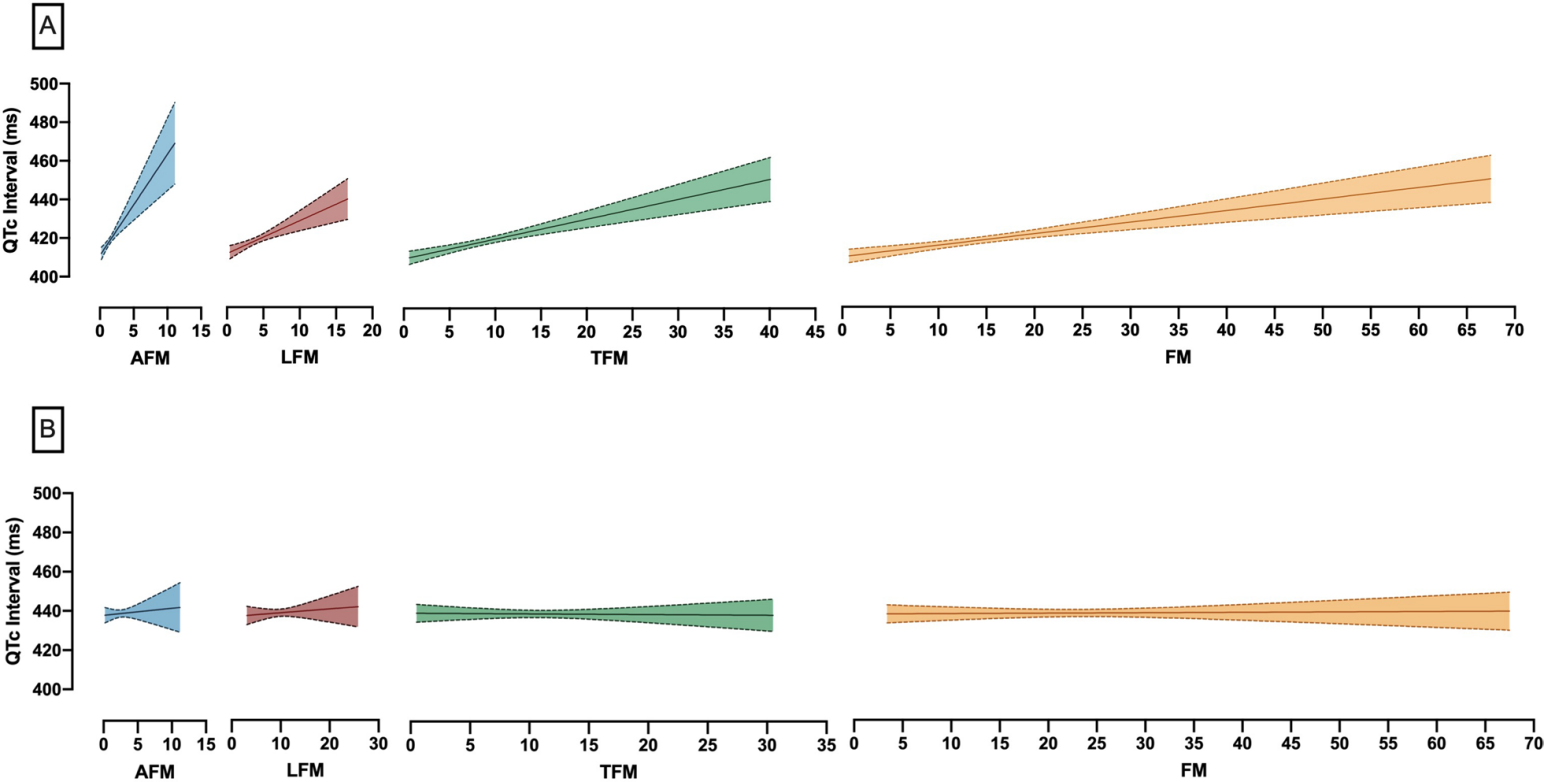
Linear regression and 95% confidence interval plot of QTc interval and fat mass in different body parts. **A** Male, **B** Female, AFM = Arm fat mass, LFM = Leg fat mass, TFM = Trunk fat mass, FM = Total fat mass, QTc interval = Corrected QT interval by Bazett formula

Surprisingly, none of the body composition variables in females did show a significant correlation with continuous QTc. In logistic regression, only four ORs remained significant after multi-variable adjusted, related to total and truncal fat percentage and FM/FFM ratio. In contrast to male results, these ORs were lower than 1.00. FM/FFM ratios in total, and trunk showed significant associations with QTc > 450 ms with ORs 0.32 (0.12–0.90), and 0.31 (0.12–0.82) respectively (P-value < 0.05). Figure 1 shows the linear regression line and 95% CI between QTc interval and fat mass in different body parts in both genders.

Additional File 2 would show the results of this study if QT intervals were corrected by Fridericia's formula. Additional File 2: Table B1 has reported that none of the body fat composition data shows significant differences between QT interval groups in men. At the same time, the mean of fat percentage, FMI, and FM/FFM ratio in total, legs and trunk had significant differences between QT interval groups in women(P-value < 0.05). Regarding the association of Fridericia's formula corrected QT interval and body composition data in male subjects, Additional File 2: Table B1 shows a lower count of significant beta-coefficients and lower beta-coefficients values than the main results (using Bazett's formula). Also, there was not any significant OR related to men (Additional File 2: Table B2). In contrast, more significant ORs were observed in female subjects, and none of the beta-coefficients of linear regressions were significant. However, both QT correction formulas showed positive and more than 1.00 OR for males and negative and less than 1.00 OR for females (Additional File 2: Table B3).

## Discussion

### Main findings

This study suggests that (i) in male contributors, there were significantly higher FM/FFM ratio in total body, arms, and trunk in prolonged QTc group compared to normal QTc group. These body composition variables, as well as FM/FFM ratio in legs, showed unequal significant positive correlations with QTc. Also, FMI of total and all regional fat had a significant positive association with QTc. (ii) in female contributors, there were substantial differences in the mean of FM/FFM ratio in trunk and legs between normal and prolonged QTc groups. Surprisingly, the normal QTc group had higher body composition variables in women. Also, none of the body composition variables had a significant correlation with continuous QTc. (iii) Fat mass ratios in the trunk, arms, total, and legs, respectively, with a higher to a lower beta-coefficient, were associated with QTc interval in males, and such associations were not observed in females.

These findings were related to a population without any electrolyte imbalance. These relations remained significant after multi-variable adjusting for age, smoking status, total cholesterol, high-density lipoprotein, and blood pressure, history of CHD, diabetes, and taking any medication that affects QT interval [25].

### Interpretation of data

To our knowledge, this is the first study to seek if different patterns of anatomical fat distribution in the body could affect the QTc interval among people; that is why we used one of the most significant databases of ECG in an Iranian population. Our study extends the knowledge from previous studies in fat mass and QTc interval by showing which part of body fat mass was related to QTc interval. Few previous studies have focused on total fat percentage, visceral and subcutaneous fat depots, upper body obesity (waist to hip circumference ratio ≥ 0.85), and BMI subjects [31–35]. It should be mentioned that the difference between the mean of BMI in normal and prolonged QTc interval groups was not significant in our study. Still, we were able to show body fat mass significant association with QTc interval in men even after multi-variable adjusting. As our study is observational, we decided to minimize other variables that may affect body fat and QTc interval by adjusting for them. Age, BP, TC, HDL, current smoking status, history of CHD, DM, HTN, and using selected medications known for prolonging QTc were the adjusting variables. It is noticeable that every significant result remained significant after multi-variable adjusting, so it can interpret that body fat mass may be an "independent risk factor" for increasing QTc, apart from conventional CVD risk factors.

The coincidence of higher QTc intervals and obesity can have a supra additive adverse effect on health. On the other hand, these two can be interrelated. Previous studies observed prolonged QTc be related to obesity and its increase [36]. Also, Braschi et al.; reported that mean of corrected QT intervals in subjects with BMI > 30 is higher than 40 ms compared to subjects with BMI < 25 [35]. With a smaller sample size, El-Gamal et al. demonstrated that relative body fat is associated with QTc interval [31]. Hillerbrand et al [32] has suggested that more visceral fat accumulation is associated with prolongation of QTc. This study was based on the magnetic resonance imaging evolution of visceral fat. We reported the same results for body fat measures and QTc in men, but our study's advantage was measuring different body parts' fat mass. This advantage enabled us to report the correlation between the different anatomic distribution of fat and QTc interval more accurately. Although body fat and QTc did not represent a significant association in women in our study, Park et al [33] and Peiris et al [34] showed a positive correlation between QTc and body fat women. The first noticeable weak point for these two studies might be their petite sample sizes (31 and 27 participants, respectively). Moreover, the method of body fat measurements is different from our method. The former study method of measurement was based on Lohman et al. calculation formula, and the second study method measured body fat mass by hydrodensitometry. Another reason may be that the female subjects in our population reported a much worse cardiovascular profile, as it was addressed before [13]. For example, the frequency of CHD in females was 11.4% compared to 5.7% in male subjects. These differences in the baseline cardiovascular characteristic of the two groups may explain the contrary findings between the two genders.

The load-capacity model in this study was utilized to identify people with insufficient muscle mass and excessive adiposity, using a tiered risk approach. The load–capacity model integrates the physiological impacts of adiposity and lean body mass components within the same individual to operationalize body composition assessment. The proportionate contribution of each component to physiological functions, which considers hormonal and age-related body composition changes, will determine risk [26]. The two indices' basic premise is that an individual's metabolic load is determined by their contribution relative to one another, not by a specific amount of the two separate components [37]. In other words, the issue is not about fat mass itself but the balance between lifestyle (with a high FM as a result of positive metabolic load) and functional capacity of the body [26]. Several previous studies have been evaluated the load–capacity model and worse health outcomes. Auyeung et al. [38] showed that FM/FFM ratio might predict a year increased risk of physical disability in the Chinese elderly. Also, it has been suggested the associations of the load–capacity model with blood pressure [39] and metabolic syndrome [40]. In this study, for the first time, the load-capacity model of total and different body regions is associated with QTc interval.

**Fig. 2:**
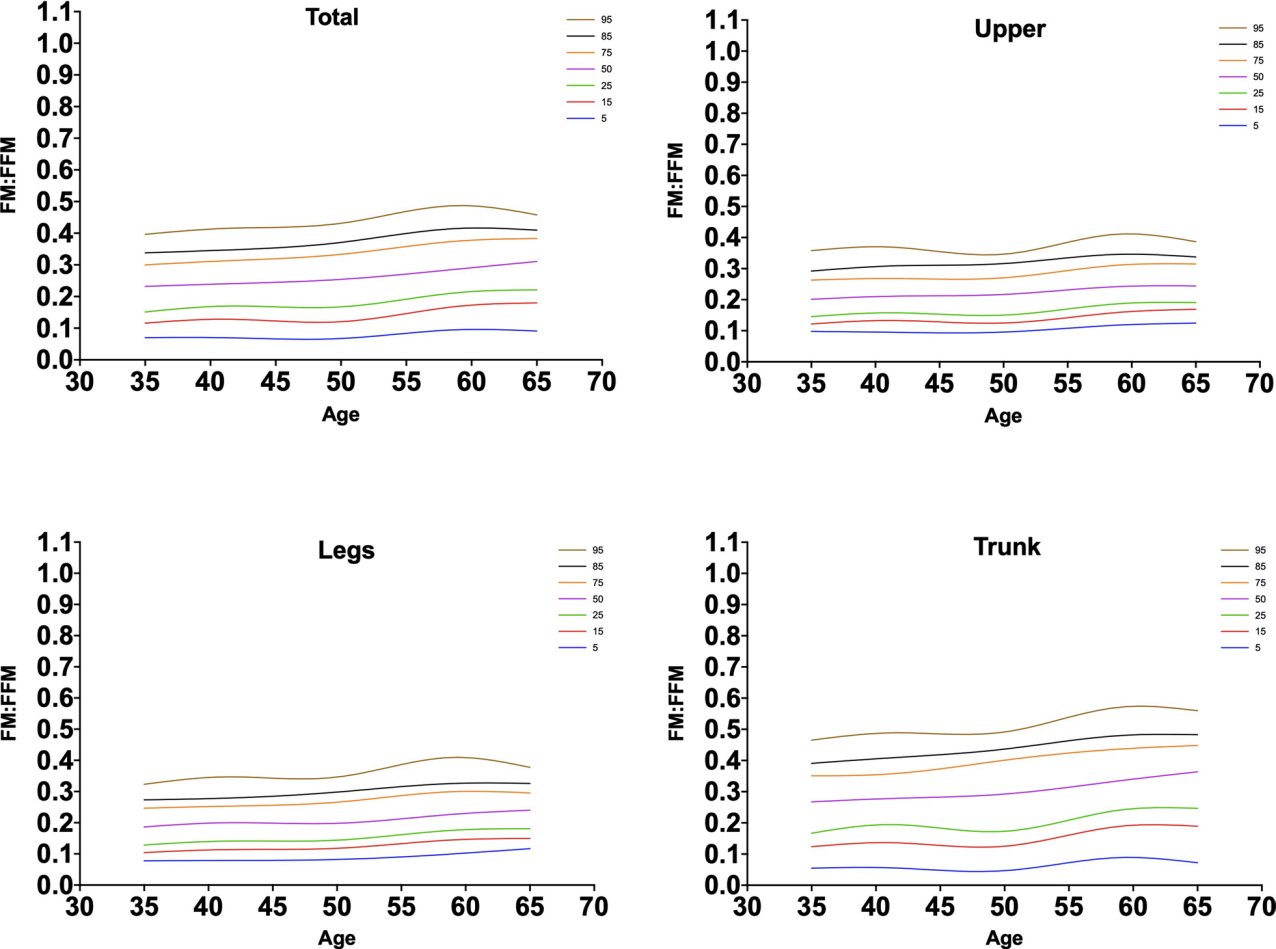
Smoothed fat mass to fat-free mass ratio percentiles for men in total and different regional fat deposits. The percentiles shown are 5th, 15th, 25th, 50th, 75th, 85th, and 95th

**Fig. 3:**
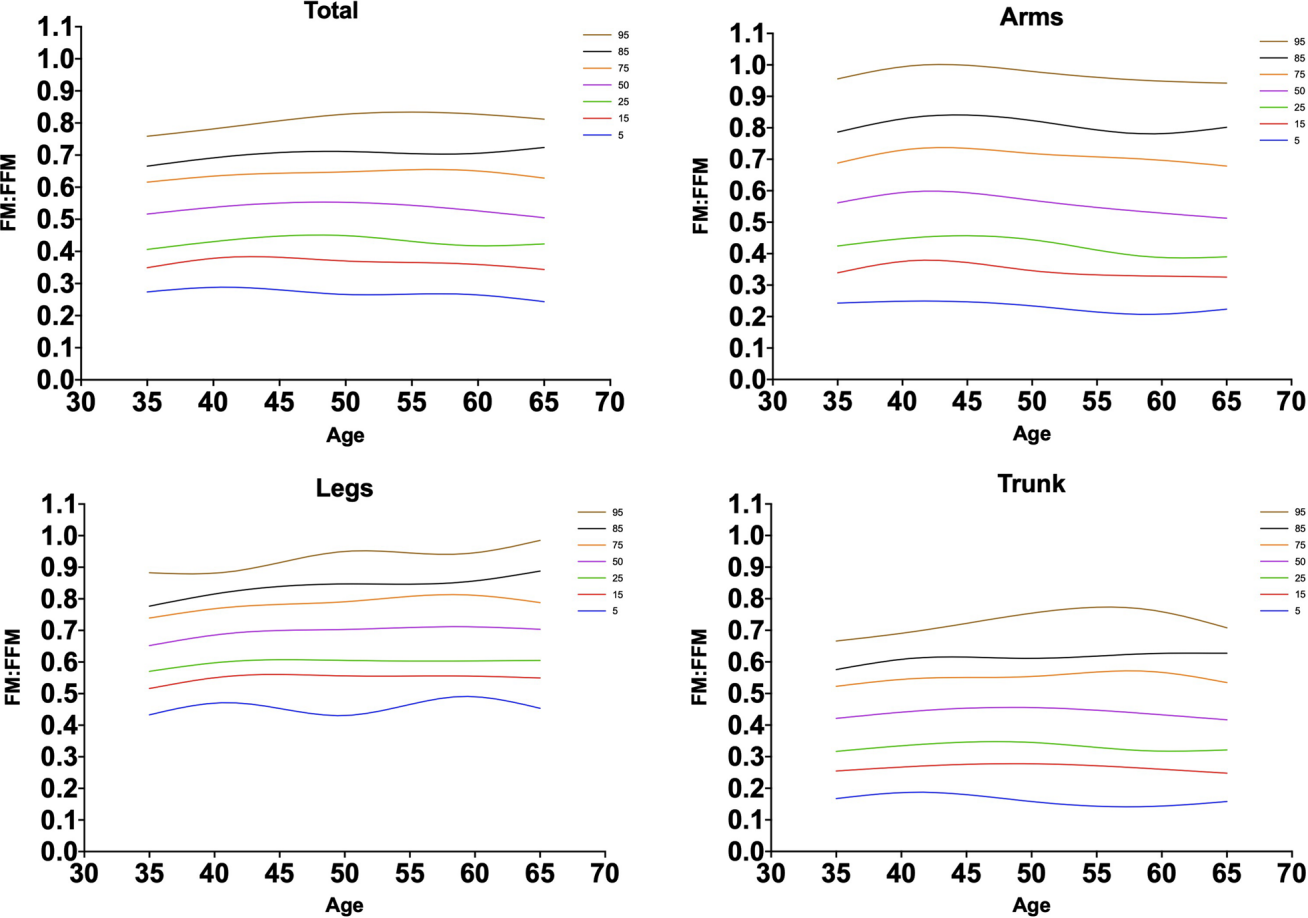
Smoothed fat mass to fat-free mass ratio percentiles for women in total and different regional fat deposits. The percentiles shown are 5th, 15th, 25th, 50th, 75th, 85th, and 95th

Comparing our data with previously published population reference values demonstrated that in all age groups, our population FM/FFM ratio values were lower than population reference values of FM/FFM ratio calculated by using both DXA [26] and BIA [41] method in both genders. Moreover, the illustration of 5th, 15th, 25th, 50th, 75th, 85th, and 95th percentiles of FM/FFM ratio in total and regional fat depots according to age have been presented in Figs. 2 and 3. In male subjects, the relationships were linear overall, which was in line with the previous literatures [26].

Several pathophysiological mechanisms could explain our findings. Upper body obesity has been reported as a risk factor for CHD, consequently prolonging the QTc interval [42]. Possible mechanisms of the prolonged QTc in obesity include autonomic nervous system alternation and repolarization abnormalities, increased cardiac output, secreted cytokines by adipose tissue, electrolyte abnormalities, increasing plasma free fatty acid (FFA) levels, insulin resistance, and hyperinsulinemia. Gao et al [43] reported that subjects with upper-body obesity had higher autonomic nervous system activity. Dominant sympathetic over parasympathetic activation leads to a higher heart rate and influences ventricular repolarization [44]. Alternation of autonomic nervous system balance can also be due to elevated stroke volume and cardiac output in obese patients [31]. Underlie of the association of obesity and fat tissue with the autonomic nervous system could be cytokine secretion by adipose tissue [45]. The secreted pro-inflammatory cytokines such as leptin can directly stimulate the central sympathetic nervous system [45–47]. As metabolic mechanisms, it can be demonstrated that prolonged QTc interval may be due to increased FFA [48, 49], insulin resistance, and hyperinsulinemia [34] in more obese individuals. Scherrer et al [50] suggested that body fat is an indicator of muscle sympathetic nerve discharge, and it is in a positive correlation with fasting insulin level. Also, Behrasi et al [51] suggested that HOMA-IR is positively correlated with body fat mass as an indicator of insulin resistance. So, it can be interpreted that more fat tissue leads to more insulin resistance and hyperinsulinemia. Another underlying mechanism of hyperinsulinemia may be due to its ion exchange role at the myocardium, which may finally induce QTc prolongation.

After all, the BIA method provided us more details about the anatomic distribution of fat through the body. Although the body composition data of total and regional parts of the body were closely intercorrelated (Additional File 1: Table A2), it was suggested that body fat mass, including trunk, arms, and legs, may be related to QTc interval unevenly. The truncal fat mass ratio had the highest value of beta-coefficient among body composition values; it may be the deposited fat in the epicardium that plays a role in the repolarization. Logically higher truncal fat indicates a higher fat deposition on the epicardial surface of the heart. This may be the underlying mechanism of prolonged repolarization with possible electrical sequelae for the myocardium. Also, some previous studies in the case of epicardial adipose tissue and QT interval reported a significant relationship between these two [52, 53]. Moreover, pericardial adipose tissue was shown to be associated with impaired fasting plasma glucose [18, 54], insulin resistance, and hypertension [19, 55]. These associations may show that pericardial adipose tissue can trigger the mechanisms mentioned above and prone subjects to CHD. Or maybe it is just a matter of ECG recording in participants with higher fat mass in the trunk? Perhaps more prolonged QT simply indicates a lag in voltage recording. This can only be measured utilizing more sophisticated devices that can record voltage changes pretty fast to exclude any delays produced by the higher fat. Of course, the extended follow-up data, when compiled, will tell us about the prognosis of this study's observations, and we will wait to see if there are higher CVD events in those with higher fat indices, mostly higher amount of FM/FFM ratios and QTc interval compared to other different body parts’ fat mass. We also suggest that this association between fat mass and QTc should investigate different variables such as electrolyte level, insulin level, FFA, physical activity, and calorie intake to minimize their influence on this relationship.

### Strengths and limitations

A strength in our study was our larger study population in comparison to other studies on this topic. Data collection of digital ECGs were done under highly standardized conditions and enabled us to report more precise ECG data. Another strength was the extensive measurements of confounding factors in the Fasa PERSIAN Cohort Study, which examined the relationship of QTc interval and fat mass apart from influential factors. Also, to our knowledge, this is the first study to seek if different patterns of anatomical fat distribution in the body have any association with the QTc interval among an Iranian population.

Our study was not free from limitations. First, as this study has a cross-sectional design, it limits our ability to infer any causality. Second, our study was based on a rural population within the age range of 35–70, which restricted us from extending our findings to a younger or urban community. Third, body composition was assessed with a BIA device. BIA systems are not considered reference methods for body composition analysis but as predictive methods with population-specific predictive algorithms. These potential errors in body composition analysis were an undeniable limitation.

At last, although the linear association of fat mass and QTc interval was strong enough, the association between fat mass and prolonged QTc interval (as a binary variable) showed a slight level of significance. So, it could not be interpreted that higher fat mass would lead to QTc intervals more than cut-points, which may be due to our small sample size in prolonged QTc interval groups. Regarding the last limitation, although the sample size of prolonged QTc interval in female subjects was reasonable compared to male subjects and 470 ms is a well-established cut point for females, another cut-point was used for female subjects in case 470 ms be a bit high (Additional File 3). Surprisingly, using a lower cut point on Bazett's formula corrected QT interval led to having zero significant difference between QT interval groups (Additional File 3: Table C1) and ORs (Additional File 3: Table C2). While using the same cut point on Fridericia's formula, corrected QT interval led to having more significant differences between QT interval groups (Additional File 3: Table C1) and ORs (Additional File 3: Table C2). These results may vary from the study's primary results, but in general, they were in line with the main results as the female subjects showed negative relationships between body composition data and QTc (even in another QT corrected formula and cut-point). For sure, longer follow-ups in our study, other and larger populations, can reveal more results in these relationships as life is more than calculating B-coefficients.

## Conclusion

Our study suggested that FM/FFM ratios in trunk, arms, total, and legs were positively associated with QTc interval in males, respectively, from a higher to a lower beta-coefficient. Our study implies that body fat mass and different body parts’ fat mass may be independent risk factors for higher QTc interval and, consequently, more cardiovascular events, which should be investigated in further studies.

## Supplementary Information

**Additional file 1.** The association between QTc interval and body fat composition data in female and the intercorrelation of total and regional body fat composition data in both genders.

**Additional file 2.** The mean of total and regional body composition data and BMI in QT interval groups and the association between QT interval, total and regional body fat composition, and fat mass index in both genders using Fridericia's formula for correcting QT intervals.

**Additional file 3.** The mean of total and regional body composition data in QTc interval groups and the association between QTc interval, total and regional body fat composition, and fat mass index in female subjects using a lower cut-point (> 460 ms).

## Data Availability

The datasets used and/or analyzed during the current study are available from the corresponding author on reasonable request to the corresponding author.
