# Supplementary material for: Different body parts’ fat mass and corrected QT interval on the electrocardiogram: The Fasa PERSIAN Cohort Study
Source: BMC Cardiovasc Disord. 2021 Jun 5;21:277. doi: 10.1186/s12872-021-02095-2 (PMC8178852; doi:10.1186/s12872-021-02095-2)
Supplement: Supplementary file 1 — Additional file 1. The association between QTc interval and body fat composition data in female and the intercorrelation of total and regional body fat composition data in both genders. [file 12872_2021_2095_MOESM1_ESM.docx]

**Table A1. The association between QTc interval and body fat composition data in female.**

|  | | **Unadjusted** | | | | **Multi-variable adjusted** | | | | | | |
| --- | --- | --- | --- | --- | --- | --- | --- | --- | --- | --- | --- | --- |
|  |  | **QTc (continuous)** | | **QTc > 470 ms** | | **QTc (continuous)** | | | **QTc > 470 ms** | | |  |
|  |  | **Beta** | **P-value** | **OR (95%CI)** | **P-value** | **Beta** | **P-value** | **OR (95%CI)** | | **P-value** |  |  |
| Total | Fat Mass (%) | -0.008 | 0.740 | **0.98 (0.96-1.00)** | **0.034** | -0.029 | 0.237 | **0.97 (0.95-1.00)** | | **0.019** |  |  |
|  | FMI (kg/m^2^) | 0.005 | 0.826 | 0.97 (0.92-1.01) | 0.153 | -0.011 | 0.645 | 0.96 (0.92-1.01) | | 0.138 |  |  |
|  | FFMI (kg/m^2^) | 0.034 | 0.144 | 1.01 (0.92-1.10) | 0.883 | 0.036 | 0.150 | 1.03 (0.94-1.13) | | 0.473 |  |  |
|  | FM:FFM Ratio | -0.008 | 0.735 | 0.39 (0.15-1.03) | 0.056 | -0.029 | 0.234 | **0.32 (0.12-0.90)** | | **0.031** |  |  |
| Arms | Fat Mass (%) | -0.005 | 0.834 | 0.99 (0.98-1.00) | 0.075 | -0.018 | 0.456 | 0.99 (0.98-1.00) | | 0.099 |  |  |
|  | FMI (kg/m^2^) | 0.012 | 0.608 | 0.85 (0.62-1.15) | 0.279 | 0.001 | 0.963 | 0.86 (0.62-1.18) | | 0.342 |  |  |
|  | FFMI (kg/m^2^) | 0.042 | 0.077 | 1.04 (0.53-2.05) | 0.910 | 0.035 | 0.159 | 1.15 (0.56-2.38) | | 0.706 |  |  |
|  | FM:FFM Ratio | -0.002 | 0.928 | 0.57 (0.28-1.16) | 0.121 | -0.015 | 0.551 | 0.58 (0.27-1.21) | | 0.145 |  |  |
| Legs | Fat Mass (%) | 0.006 | 0.814 | 0.99 (0.98-1.00) | 0.155 | -0.023 | 0.354 | 0.98 (0.97-1.00) | | 0.047 |  |  |
|  | FMI (kg/m^2^) | 0.015 | 0.523 | 0.94 (0.82-1.07) | 0.340 | -0.002 | 0.938 | 0.93 (0.81-1.07) | | 0.290 |  |  |
|  | FFMI (kg/m^2^) | 0.024 | 0.303 | 0.96 (0.77-1.21) | 0.745 | 0.030 | 0.224 | 1.06 (0.83-1.36) | | 0.628 |  |  |
|  | FM:FFM Ratio | 0.005 | 0.827 | 0.48 (0.16-1.42) | 0.183 | -0.024 | 0.339 | 0.32 (0.10-1.01) | | 0.052 |  |  |
| Trunk | Fat Mass (%) | -0.014 | 0.550 | **0.98 (0.96-1.00)** | **0.018** | -0.033 | 0.179 | **0.98 (0.96-1.00)** | | **0.012** |  |  |
|  | FMI (kg/m^2^) | -0.004 | 0.878 | 0.93 (0.85-1.01) | 0.077 | -0.021 | 0.400 | 0.92 (0.84-1.01) | | 0.066 |  |  |
|  | FFMI (kg/m^2^) | 0.037 | 0.112 | 1.04 (0.89-1.21) | 0.658 | 0.037 | 0.135 | 1.08 (0.91-1.27) | | 0.386 |  |  |
|  | FM:FFM Ratio | -0.016 | 0.497 | **0.35 (0.14-0.90)** | **0.030** | -0.035 | 0.151 | **0.31 (0.12-0.82)** | | **0.019** |  |  |

QTc= Corrected QT interval by Bazett formula, OR= Odds ratio, CI= Confidence interval. Statistically significant P-values are bolded.

**Table A2. The intercorrelation of total and regional body fat composition data in both genders.**

| Male  Female | | Total | | | | Arms | | | | Legs | | | | Trunk | | | |
| --- | --- | --- | --- | --- | --- | --- | --- | --- | --- | --- | --- | --- | --- | --- | --- | --- | --- |
|  |  | % | FMI | FFMI | FM:FFM | % | FMI | FFMI | FM:FFM | % | FMI | FFMI | FM:FFM | % | FMI | FFMI | FM:FFM |
| Total | % |  | 0.957 | 0.569 | 0.993 | 0.956 | 0.851 | 0.535 | 0.927 | 0.939 | 0.900 | 0.613 | 0.922 | 0.990 | 0.969 | 0.519 | 0.984 |
|  | FMI | 0.947 |  | 0.720 | 0.973 | 0.966 | 0.959 | 0.687 | 0.962 | 0.931 | 0.968 | 0.760 | 0.927 | 0.933 | 0.993 | 0.658 | 0.954 |
|  | FFMI | 0.525 | 0.717 |  | 0.576 | 0.661 | 0.793 | 0.971 | 0.647 | 0.641 | 0.762 | 0.957 | 0.625 | 0.516 | 0.673 | 0.974 | 0.528 |
|  | FM:FFM | 0.989 | 0.966 | 0.531 |  | 0.967 | 0.884 | 0.542 | 0.950 | 0.943 | 0.923 | 0.624 | 0.936 | 0.979 | 0.981 | 0.520 | 0.989 |
| Arms | % | 0.950 | 0.961 | 0.699 | 0.944 |  | 0.926 | 0.586 | 0.987 | 0.904 | 0.916 | 0.713 | 0.897 | 0.937 | 0.967 | 0.607 | 0.952 |
|  | FMI | 0.860 | 0.973 | 0.810 | 0.889 | 0.934 |  | 0.747 | 0.952 | 0.841 | 0.941 | 0.839 | 0.847 | 0.820 | 0.936 | 0.721 | 0.861 |
|  | FFMI | 0.606 | 0.769 | 0.950 | 0.611 | 0.698 | 0.820 |  | 0.574 | 0.629 | 0.747 | 0.923 | 0.614 | 0.480 | 0.635 | 0.933 | 0.492 |
|  | FM:FFM | 0.921 | 0.980 | 0.716 | 0.941 | 0.978 | 0.975 | 0.711 |  | 0.880 | 0.916 | 0.708 | 0.882 | 0.906 | 0.957 | 0.586 | 0.936 |
| Legs | % | 0.930 | 0.919 | 0.610 | 0.923 | 0.916 | 0.865 | 0.673 | 0.897 |  | 0.956 | 0.610 | 0.994 | 0.885 | 0.908 | 0.633 | 0.888 |
|  | FMI | 0.876 | 0.973 | 0.814 | 0.896 | 0.936 | 0.984 | 0.838 | 0.962 | 0.923 |  | 0.760 | 0.965 | 0.846 | 0.933 | 0.721 | 0.871 |
|  | FFMI | 0.619 | 0.788 | 0.949 | 0.630 | 0.776 | 0.863 | 0.902 | 0.796 | 0.606 | 0.835 |  | 0.598 | 0.588 | 0.728 | 0.870 | 0.607 |
|  | FM:FFM | 0.925 | 0.940 | 0.625 | 0.934 | 0.916 | 0.896 | 0.688 | 0.918 | 0.992 | 0.945 | 0.626 |  | 0.864 | 0.899 | 0.613 | 0.875 |
| Trunk | % | 0.981 | 0.895 | 0.411 | 0.967 | 0.895 | 0.784 | 0.512 | 0.860 | 0.847 | 0.784 | 0.542 | 0.842 |  | 0.961 | 0.447 | 0.991 |
|  | FMI | 0.974 | 0.980 | 0.595 | 0.990 | 0.942 | 0.915 | 0.674 | 0.949 | 0.890 | 0.908 | 0.700 | 0.908 | 0.957 |  | 0.605 | 0.978 |
|  | FFMI | 0.407 | 0.607 | 0.975 | 0.410 | 0.598 | 0.715 | 0.908 | 0.611 | 0.557 | 0.737 | 0.859 | 0.567 | 0.271 | 0.465 |  | 0.452 |
|  | FM:FFM | 0.970 | 0.911 | 0.410 | 0.980 | 0.888 | 0.806 | 0.507 | 0.879 | 0.841 | 0.799 | 0.546 | 0.851 | 0.986 | 0.972 | 0.267 |  |

Data presented as Pearson correlation coefficient. %: Fat mass in percentage, FMI: Fat Mass Index, FFMI: Fat-Free Mass Index
