# Supplementary material for: Different body parts’ fat mass and corrected QT interval on the electrocardiogram: The Fasa PERSIAN Cohort Study
Source: BMC Cardiovasc Disord. 2021 Jun 5;21:277. doi: 10.1186/s12872-021-02095-2 (PMC8178852; doi:10.1186/s12872-021-02095-2)
Supplement: Supplementary file 2 — Additional file 2. The mean of total and regional body composition data and BMI in QT interval groups and the association between QT interval, total and regional body fat composition, and fat mass index in both genders using Fridericia's formula for correcting QT intervals. [file 12872_2021_2095_MOESM2_ESM.docx]

**Table B1. The mean and standard deviation of total and regional body composition data and BMI in normal and prolonged corrected QT interval by Fridericia's formula groups according to gender.**

|  | | **Male** | | | **Female** | | |
| --- | --- | --- | --- | --- | --- | --- | --- |
|  |  | **QTc ≤ 450ms**  **(n=1332)** | **QTc > 450ms**  **(n=83)** | **p-value** | **QTc ≤ 470ms**  **(n=1717)** | **QTc > 470ms**  **(n=85)** | **p-value** |
| Total | Fat Mass (%) | 19.50±7.10 | 19.90±7.40 | 0.555 | 34.20±6.90 | 32.30±7.70 | **0.016** |
|  | FMI (kg/m^2^) | 4.97±2.57 | 5.05±2.41 | 0.778 | 9.61±3.46 | 8.74±3.33 | **0.024** |
|  | FFMI (kg/m^2^) | 19.18±2.28 | 19.07±1.92 | 0.657 | 17.62±1.80 | 17.38±1.71 | 0.215 |
|  | FM:FFM Ratio | 0.25±0.11 | 0.26±0.11 | 0.522 | 0.54±0.16 | 0.50±0.16 | **0.022** |
| Arms | Fat Mass (%) | 35.55±11.44 | 36.84±11.26 | 0.321 | 70.99±18.11 | 65.91±18.94 | **0.012** |
|  | FMI (kg/m^2^) | 0.50±0.27 | 0.51±0.23 | 0.830 | 1.07±0.53 | 0.95±0.47 | **0.028** |
|  | FFMI (kg/m^2^) | 2.15±0.36 | 2.12±0.32 | 0.391 | 1.78±0.23 | 1.76±0.22 | 0.304 |
|  | FM:FFM Ratio | 0.22±0.09 | 0.23±0.09 | 0.338 | 0.58±0.22 | 0.52±0.21 | **0.015** |
| Legs | Fat Mass (%) | 33.57±11.50 | 33.74±11.49 | 0.897 | 80.74±10.14 | 78.77±10.37 | **0.080** |
|  | FMI (kg/m^2^) | 1.40±0.71 | 1.40±0.66 | 0.925 | 4.07±1.20 | 3.82±1.11 | 0.052 |
|  | FFMI (kg/m^2^) | 6.53±0.87 | 6.52±0.74 | 0.911 | 5.83±0.68 | 5.69±0.62 | 0.057 |
|  | FM:FFM Ratio | 0.21±0.09 | 0.21±0.08 | 0.940 | 0.69±0.14 | 0.66±0.14 | 0.077 |
| Trunk | Fat Mass (%) | 21.40±8.30 | 22.10±8.80 | 0.468 | 29.70±8.30 | 27.40±9.30 | **0.012** |
|  | FMI (kg/m^2^) | 3.08±1.62 | 3.16±1.56 | 0.655 | 4.47±1.81 | 3.99±1.81 | **0.017** |
|  | FFMI (kg/m^2^) | 10.50±1.13 | 10.43±0.97 | 0.603 | 10.02±0.97 | 9.94±0.92 | 0.453 |
|  | FM:FFM Ratio | 0.29±0.14 | 0.30±0.14 | 0.410 | 0.44±0.17 | 0.40±0.17 | **0.018** |

Data presented as Mean±Standard deviation. QTc= corrected QT interval by Fridericia's formula. P-value reported as the result of independent-samples t-test between normal and prolonged QTc interval groups. Statistically significant P-values are bolded(P-value<0.05).

**Table B2. The association between QT interval corrected by Fridericia's formula, total and regional body fat composition and fat mass index in male**

|  | | **Unadjusted** | | | | | **Multi-variable adjusted** | | | | | | |
| --- | --- | --- | --- | --- | --- | --- | --- | --- | --- | --- | --- | --- | --- |
|  |  | **QTc (continuous)** | | **QTc > 450 ms** | | **QTc (continuous)** | | | | **QTc > 450 ms** | | |  |
|  |  | **Beta** | **P-value** | **OR (95%CI)** | **P-value** | **Beta** | | **P-value** | **OR (95%CI)** | | **P-value** |  |  |
| Total | Fat Mass (%) | **0.064** | **0.016** | 1.01 (0.98-1.04) | 0.555 | **0.066** | | **0.028** | 1.03 (0.98-1.08) | | 0.298 |  |  |
|  | FMI (kg/m^2^) | **0.055** | **0.039** | 1.01 (0.93-1.10) | 0.778 | **0.059** | | **0.049** | 1.03 (0.90-1.18) | | 0.649 |  |  |
|  | FFMI (kg/m^2^) | -0.013 | 0.632 | 0.98 (0.89-1.08) | 0.657 | 0.001 | | 0.970 | 0.96 (0.81-1.12) | | 0.581 |  |  |
|  | FM:FFM Ratio | **0.068** | **0.011** | 1.92 (0.26-14.10) | 0.521 | **0.069** | | **0.022** | 4.43 (0.19-104.35) | | 0.356 |  |  |
| Arms | Fat Mass (%) | **0.081** | **0.002** | 1.01 (0.99-1.03) | 0.321 | **0.088** | | **0.003** | 1.02 (0.99-1.05) | | 0.181 |  |  |
|  | FMI (kg/m^2^) | 0.046 | 0.081 | 1.09 (0.49-2.45) | 0.830 | 0.055 | | 0.059 | 1.23 (0.35-4.36) | | 0.752 |  |  |
|  | FFMI (kg/m^2^) | -0.042 | 0.112 | 0.76 (0.40-1.43) | 0.390 | -0.022 | | 0.463 | 9.09 (0.26-314.85) | | 0.222 |  |  |
|  | FM:FFM Ratio | **0.080** | **0.003** | 3.13 (0.30-32.28) | 0.337 | **0.084** | | **0.004** | 0.96 (0.57-1.62) | | 0.879 |  |  |
| Legs | Fat Mass (%) | 0.030 | 0.263 | 1.00 (0.98-1.02) | 0.896 | 0.024 | | 0.436 | 1.03 (0.68-1.56) | | 0.879 |  |  |
|  | FMI (kg/m^2^) | 0.028 | 0.289 | 0.99 (0.72-1.35) | 0.925 | 0.032 | | 0.276 | 0.89 (0.01-60.56) | | 0.955 |  |  |
|  | FFMI (kg/m^2^) | 0.004 | 0.868 | 0.99 (0.76-1.28) | 0.911 | 0.038 | | 0.206 | 1.03 (0.99-1.08) | | 0.175 |  |  |
|  | FM:FFM Ratio | 0.032 | 0.236 | 1.10 (0.08-14.50) | 0.940 | 0.025 | | 0.399 | 1.08 (0.88-1.34) | | 0.470 |  |  |
| Trunk | Fat Mass (%) | **0.074** | **0.005** | 1.01 (0.98-1.04) | 0.468 | **0.077** | | **0.009** | 0.87 (0.63-1.19) | | 0.388 |  |  |
|  | FMI (kg/m^2^) | **0.066** | **0.013** | 1.03 (0.90-1.18) | 0.655 | **0.068** | | **0.023** | 5.19 (0.42-64.12) | | 0.199 |  |  |
|  | FFMI (kg/m^2^) | -0.015 | 0.577 | 0.95 (0.78-1.16) | 0.602 | -0.018 | | 0.544 | 1.03 (0.98-1.08) | | 0.298 |  |  |
|  | FM:FFM Ratio | **0.079** | **0.003** | 1.98 (0.39-10.06) | 0.410 | **0.081** | | **0.006** | 1.03 (0.90-1.18) | | 0.649 |  |  |

QTc= corrected QT interval by Fridericia's formula. OR= Odds ratio, CI= Confidence interval. Statistically significant P-values are bolded(P-value<0.05).

**Table B3. The association between QT interval corrected by Fridericia's formula, total and regional body fat composition and fat mass index in female**

|  | | **Unadjusted** | | | | **Multi-variable adjusted** | | | | | | |
| --- | --- | --- | --- | --- | --- | --- | --- | --- | --- | --- | --- | --- |
|  |  | **QTc (continuous)** | | **QTc > 470 ms** | | **QTc (continuous)** | | | **QTc > 470 ms** | | |  |
|  |  | **Beta** | **P-value** | **OR (95%CI)** | **P-value** | **Beta** | **P-value** | **OR (95%CI)** | | **P-value** |  |  |
| Total | Fat Mass (%) | -0.032 | 0.173 | **0.96 (0.94-0.99)** | **0.016** | -0.038 | 0.123 | **0.96 (0.93-0.99)** | | **0.014** |  |  |
|  | FMI (kg/m^2^) | -0.024 | 0.313 | **0.93 (0.87-0.990** | **0.024** | -0.024 | 0.321 | **0.93 (0.86-0.99)** | | **0.033** |  |  |
|  | FFMI (kg/m^2^) | 0.008 | 0.728 | 0.92 (0.82-1.05) | 0.215 | 0.025 | 0.305 | 0.97 (0.85-1.11) | | 0.638 |  |  |
|  | FM:FFM Ratio | -0.033 | 0.161 | **0.20 (0.05-0.79)** | **0.022** | -0.040 | 0.106 | **0.17 (0.04-0.74)** | | **0.018** |  |  |
| Arms | Fat Mass (%) | -0.033 | 0.164 | **0.98 (0.97-0.99)** | **0.012** | -0.030 | 0.226 | **0.99 (0.98-1.00)** | | **0.029** |  |  |
|  | FMI (kg/m^2^) | -0.018 | 0.442 | 0.59 (0.37-0.94) | 0.028 | -0.013 | 0.585 | 0.62 (0.38-1.02) | | 0.059 |  |  |
|  | FFMI (kg/m^2^) | 0.016 | 0.510 | 0.60 (0.23-1.59) | 0.304 | 0.026 | 0.294 | 0.74 (0.26-2.10) | | 0.571 |  |  |
|  | FM:FFM Ratio | -0.030 | 0.196 | **0.27 (0.10-0.78)** | **0.015** | -0.028 | 0.257 | **0.30 (0.10-0.90)** | | **0.031** |  |  |
| Legs | Fat Mass (%) | -0.017 | 0.480 | 0.98 (0.96-1.00) | 0.080 | -0.031 | 0.219 | **0.98 (0.96-1.00)** | | **0.035** |  |  |
|  | FMI (kg/m^2^) | -0.014 | 0.543 | 0.82 (0.68-1.00) | 0.051 | -0.016 | 0.527 | 0.82 (0.67-1.01) | | 0.064 |  |  |
|  | FFMI (kg/m^2^) | -0.007 | 0.767 | 0.72 (0.51-1.01) | 0.057 | 0.015 | 0.562 | 0.84 (0.59-1.20) | | 0.342 |  |  |
|  | FM:FFM Ratio | -0.018 | 0.433 | 0.24 (0.05-1.16) | 0.077 | -0.033 | 0.179 | **0.17 (0.03-0.84)** | | **0.030** |  |  |
| Trunk | Fat Mass (%) | -0.036 | 0.124 | **0.97 (0.95-0.99)** | **0.012** | -0.041 | 0.099 | **0.97 (0.94-0.99)** | | **0.012** |  |  |
|  | FMI (kg/m^2^) | -0.030 | 0.197 | **0.86 (0.76-0.97)** | **0.018** | -0.032 | 0.193 | **0.86 (0.75-0.98)** | | **0.022** |  |  |
|  | FFMI (kg/m^2^) | 0.016 | 0.489 | 0.92 (0.73-1.15) | 0.452 | 0.030 | 0.214 | 0.99 (0.78-1.26) | | 0.922 |  |  |
|  | FM:FFM Ratio | -0.038 | 0.103 | **0.20 (0.05-0.76)** | **0.018** | -0.044 | 0.074 | **0.18 (0.05-0.74)** | | **0.017** |  |  |

QTc= corrected QT interval by Fridericia's formula. OR= Odds ratio, CI= Confidence interval. Statistically significant P-values are bolded(P-value<0.05).
