# Supplementary material for: Different body parts’ fat mass and corrected QT interval on the electrocardiogram: The Fasa PERSIAN Cohort Study
Source: BMC Cardiovasc Disord. 2021 Jun 5;21:277. doi: 10.1186/s12872-021-02095-2 (PMC8178852; doi:10.1186/s12872-021-02095-2)
Supplement: Supplementary file 3 — Additional file 3. The mean of total and regional body composition data in QTc interval groups and the association between QTc interval, total and regional body fat composition, and fat mass index in female subjects using a lower cut-point (> 460 ms). [file 12872_2021_2095_MOESM3_ESM.docx]

|  | | Corrected by Bazett's formula | | | Corrected by Fridericia's formula | | |
| --- | --- | --- | --- | --- | --- | --- | --- |
|  |  | **QTc ≤ 460ms**  **(n=1505)** | **QTc > 460ms**  **(n=294)** | **P-value** | **QTc ≤ 460ms**  **(n=1673)** | **QTc > 460ms**  **(n=129)** | **P-value** |
| Total | Fat Mass (%) | 34.20±6.90 | 33.70±7.40 | 0.268 | **34.30±6.90** | **32.20±7.50** | **0.001** |
|  | FMI (kg/m^2^) | 9.58±3.43 | 9.48±3.59 | 0.645 | **9.64±3.46** | **8.68±3.26** | **0.003** |
|  | FFMI (kg/m^2^) | 17.60±1.79 | 17.67±1.84 | 0.546 | 17.63±1.80 | 17.41±1.75 | 0.179 |
|  | FM:FFM Ratio | 0.54±0.16 | 0.53±0.17 | 0.354 | **0.54±0.16** | **0.49±0.16** | **0.001** |
| Arms | Fat Mass (%) | 70.91±17.95 | 69.95±19.32 | 0.408 | **71.13±18.10** | **65.80±18.56** | **0.001** |
|  | FMI (kg/m^2^) | 1.07±0.52 | 1.06±0.54 | 0.901 | **1.08±0.53** | **0.94±0.46** | **0.005** |
|  | FFMI (kg/m^2^) | 1.78±0.23 | 1.79±0.23 | 0.496 | 1.78±0.23 | 1.76±0.22 | 0.225 |
|  | FM:FFM Ratio | 0.58±0.22 | 0.57±0.23 | 0.557 | **0.58±0.22** | **0.52±0.21** | **0.001** |
| Legs | Fat Mass (%) | 80.74±10.01 | 80.17±10.87 | 0.373 | **80.83±10.10** | **78.29±10.59** | **0.006** |
|  | FMI (kg/m^2^) | 4.06±1.18 | 4.05±1.25 | 0.880 | **4.08±1.20** | **3.79±1.10** | **0.008** |
|  | FFMI (kg/m^2^) | 5.82±0.67 | 5.84±0.70 | 0.543 | 5.83±0.68 | 5.71±0.64 | 0.049 |
|  | FM:FFM Ratio | 0.69±0.14 | 0.68±0.15 | 0.471 | **0.69±0.14** | **0.66±0.14** | **0.006** |
| Trunk | Fat Mass (%) | 29.70±8.30 | 29.10±8.70 | 0.236 | **29.80±8.30** | **27.20±8.90** | **0.001** |
|  | FMI (kg/m^2^) | 4.46±1.81 | 4.38±1.86 | 0.460 | **4.49±1.82** | **3.96±1.76** | **0.002** |
|  | FFMI (kg/m^2^) | 10.01±0.96 | 10.05±0.96 | 0.601 | 10.02±0.97 | 9.95±0.93 | 0.402 |
|  | FM:FFM Ratio | 0.44±0.17 | 0.43±0.17 | 0.273 | **0.44±0.17** | **0.39±0.16** | **0.001** |

**Table C1. The mean and standard deviation of total and regional body composition data in normal QTc and prolonged QTc interval groups in female.**

Data presented as Mean±Standard deviation. P-value reported as the result of independent-samples t-test between normal and prolonged QTc interval groups. Statistically significant P-values are bolded(P-value<0.05).

**Table C2. The association between QTc interval, total and regional body fat composition and fat mass index in female**

|  | | **Corrected by Bazett's formula (QTc > 460 ms)** | | | | **Corrected by Fridericia's formula (QTc > 460 ms)** | | | |
| --- | --- | --- | --- | --- | --- | --- | --- | --- | --- |
|  |  | **Unadjusted** | | **Multi-variable adjusted** | | **Unadjusted** | | **Multi-variable adjusted** | |
|  |  | **OR (95%CI)** | **P-value** | **OR (95%CI)** | **P-value** | **OR (95%CI)** | **P-value** | **OR (95%CI)** | **P-value** |
| Total | Fat Mass (%) | 0.99 (0.97-1.01) | 0.268 | 0.99 (0.97-1.00) | 0.120 | **0.96 (0.94-0.98)** | **0.001** | **0.96 (0.93-0.98)** | **0.001** |
|  | FMI (kg/m^2^) | 0.99 (0.96-1.03) | 0.645 | 0.99 (0.95-1.03) | 0.482 | **0.92 (0.87-0.97)** | **0.003** | **0.92 (0.86-0.97)** | **0.004** |
|  | FFMI (kg/m^2^) | 1.02 (0.95-1.10) | 0.546 | 1.04 (0.97-1.12) | 0.280 | 0.93 (0.84-1.03) | 0.179 | 0.98 (0.88-1.09) | 0.645 |
|  | FM:FFM Ratio | 0.69 (0.31-1.51) | 0.354 | 0.56 (0.24-1.27) | 0.163 | **0.15 (0.05-0.48)** | **0.001** | **0.13 (0.04-0.43)** | **0.001** |
| Arms | Fat Mass (%) | 1.00 (0.99-1.00) | 0.407 | 1.00 (0.99-1.00) | 0.373 | **0.99 (0.98-0.99)** | **0.001** | **0.99 (0.98-1.00)** | **0.004** |
|  | FMI (kg/m^2^) | 0.99 (0.78-1.25) | 0.901 | 0.98 (0.76-1.27) | 0.889 | **0.57 (0.39-0.85)** | **0.005** | **0.60 (0.40-0.90)** | **0.013** |
|  | FFMI (kg/m^2^) | 1.21 (0.70-2.10) | 0.496 | 1.27 (0.71-2.30) | 0.419 | 0.61 (0.27-1.36) | 0.225 | 0.74 (0.32-1.75) | 0.498 |
|  | FM:FFM Ratio | 0.85 (0.48-1.48) | 0.557 | 0.82 (0.45-1.48) | 0.510 | **0.25 (0.10-0.58)** | **0.001** | **0.26 (0.11-0.65)** | **0.004** |
| Legs | Fat Mass (%) | 0.99 (0.98-1.01) | 0.373 | 0.99 (0.98-1.00) | 0.089 | **0.98 (0.96-0.99)** | **0.006** | **0.97 (0.96-0.99)** | **0.002** |
|  | FMI (kg/m^2^) | 0.99 (0.89-1.10) | 0.880 | 0.98 (0.87-1.09) | 0.665 | **0.80 (0.68-0.94)** | **0.008** | **0.80 (0.67-0.95)** | **0.010** |
|  | FFMI (kg/m^2^) | 1.06 (0.88-1.27) | 0.543 | 1.14 (0.94-1.39) | 0.181 | **0.76 (0.58-1.00)** | **0.049** | 0.88 (0.66-1.18) | 0.390 |
|  | FM:FFM Ratio | 0.72 (0.30-1.76) | 0.471 | 0.47 (0.18-1.22) | 0.120 | **0.17 (0.05-0.61)** | **0.007** | **0.11 (0.03-0.42)** | **0.001** |
| Trunk | Fat Mass (%) | 0.99 (0.98-1.01) | 0.236 | 0.99 (0.97-1.00) | 0.121 | **0.97 (0.95-0.99)** | **0.001** | **0.96 (0.94-0.99)** | **0.001** |
|  | FMI (kg/m^2^) | 0.97 (0.91-1.04) | 0.460 | 0.96 (0.90-1.04) | 0.314 | **0.85 (0.77-0.94)** | **0.002** | **0.84 (0.76-0.94)** | **0.002** |
|  | FFMI (kg/m^2^) | 1.04 (0.91-1.18) | 0.601 | 1.06 (0.93-1.22) | 0.388 | 0.92 (0.76-1.11) | 0.402 | 0.99 (0.81-1.21) | 0.925 |
|  | FM:FFM Ratio | 0.66 (0.31-1.40) | 0.273 | 0.55 (0.25-1.21) | 0.137 | **0.16 (0.05-0.49)** | **0.001** | **0.14 (0.05-0.45)** | **0.001** |

OR= Odds ratio, CI= Confidence interval. Statistically significant P-values are bolded.
